# Thickness and Wavelength Optimizations of a High-Performance SPR Sensor Employing a Silver Layer and Black Phosphorus in Principal Directions

**DOI:** 10.3390/nano15110790

**Published:** 2025-05-24

**Authors:** Jakub Chylek, Dalibor Ciprian, Petr Hlubina

**Affiliations:** Department of Physics, Technical University Ostrava, 17. listopadu 2172/15, 70800 Ostrava-Poruba, Czech Republic; jakub.chylek@vsb.cz (J.C.);

**Keywords:** SPR, angular domain, silver layer, black phosphorus, zigzag and armchair directions, sensitivity enhancement

## Abstract

In this paper, we propose an innovative approach based on the wavelength optimization of a light source for a simple, high-performance surface plasmon resonance (SPR) sensor utilizing comprehensive reflectance analysis in the angular domain. The proposed structure consists of a glass substrate, an adhesion layer of titanium dioxide, a silver plasmonic layer, and a 2D material. Analysis is performed in the Kretschmann configuration for liquid analyte sensing. Sensing parameters such as the refractive index (RI) sensitivity, the reflectance minimum, and the figure of merit (FOM) are investigated in the first step of this study as a function of the thickness of the silver layer together with the RI of a coupling prism. Next, utilizing the results offering a fused silica prism, the thickness of the silver layer and the wavelength of the light source are optimized for the structure with the addition of a 2D material, black phosphorus (BP), which is studied along different principal directions, the zigzag and armchair directions. In addition, a new approach of adjusting the source wavelength using a one-dimensional photonic crystal combined with an LED, is presented. Based on this analysis, for the reference structure at a wavelength of 632.8 nm, the optimized silver layer thickness is 50 nm, and the achieved RI sensitivity ranges from 193.9 to 251.5 degrees per RI unit (deg/RIU), with the highest FOM reaching 52.3 RIU^−1^. In addition, for the modified structure with BP, the achieved RI sensitivity varies in the range of 269.1–351.2 deg/RIU at the optimized wavelength of 628 nm, with the highest FOM reaching 44.7 RIU^−1^ for the zigzag direction. Due to the optimization and adjusting the wavelength of the source, the results obtained for the proposed SPR structure could have significant implications for the development of more sensitive and efficient sensors employing a simple plasmonic structure.

## 1. Introduction

Surface plasmons (SPs) are optically induced collective oscillations of free electrons at the surface of thin metal films and possess a number of beneficial properties for photonic technologies [1]. Such structures exhibit strong interaction with light accompanied by significant optical enhancements, which result in near-field intensities remarkably larger than the incident light intensity. Upon external illumination SPs cannot be directly excited; rather, a type of a coupling element is needed (a coupling prism is the usual choice). When a coupling prism in the Kretschmann configuration [2] is employed and light is directed to the interface with a particular angle of incidence, the surface plasmon resonance (SPR) phenomenon occurs. This effect can be used in various scientific fields, particularly in sensing, due to its high sensitivity to the refractive index (RI) change in surrounding media [3,4,5]. Both intensity [6] and phase [7] detections in SPR sensing utilize spectral [8,9,10] or angular interrogation techniques [10,11].

Various metals have been used in SPR sensing, of which gold (Au) and silver (Ag) are the most widely employed [4]. Gold is preferred due to its resistance to oxidation and good chemical stability. On the other hand, silver is prone to oxidation [3], but due to its very high real part of the dielectric constant, it has a narrower resonance curve and has a higher resolution compared to Au.

To enhance the performance of SPR sensors in the angular domain, different approaches have been employed. The most effective ones include using bimetallic layers [12] or 2D materials [13] such as graphene [14,15,16], black phosphorus (BP) [17,18,19,20,21,22,23,24,25,26], metal dichalcogenides [27,28,29], and franckeite [30]. Two-dimensional materials are advantageous due to their large specific area and excellent adsorption properties towards some biomolecules, especially those with carbon-based ring structures (like ssDNA) that are suitable for graphene [31]. Currently, BP is receiving significant attention because its properties outperform those of graphene in some cases, but BP is characterized by environmental instability [32] and needs some kind of passivation [33].

Another approach to enhance the performance of SPR sensors, especially their RI sensitivity, is based on complex structures whose parameters significantly affect sensing capabilities. Therefore, carefully selecting the plasmonic materials and architectural features of SPR structures is pivotal in enhancing the sensor’s performance [12,13]. Moreover, further research has been published in which the selection of proper 2D material composition [13] has been comprehensively analyzed, and employing graphene [14,15,16] or BP [17,18,19,20,21,22,23,24,25,26,33] has led to significant improvement in performance compared to conventional SPR biosensors. In addition, BP enhances light-matter interaction via the strong coupling between BP and the optical evanescent field in the total internal reflection mode [34,35,36,37]. Having established the role of BP in sensitivity enhancement, we now address the synergistic optimization of wavelength and structural parameters in maximizing sensor performance.

Generally, RI sensitivity enhancement is possible through choosing a proper prism [13] in the Kretschmann configuration or varying the wavelength of incident light [4,38,39,40,41,42,43,44] via employing a variable-wavelength source such as a white-light one with either a monochromator [39] or an acousto-optic tunable filter [43]. However, these sources can be too bulky [39] or their spectra can be too wide [43]. Recently we proposed and realized narrow-linewidth LED-based sources [45] employing a one-dimensional photonic crystal (1DPhC) with a defect layer whose tunability can range from approximately 612 nm up to 625.4 nm and from 672 up to 697.7 nm, respectively.

In this paper, we present a simple design for a high-performance SPR sensor. The proposed SPR structure comprises a glass substrate, a titanium dioxide adhesion layer, a silver plasmonic layer, and a 2D material. To achieve ideal sensor performance, we optimized the sensor parameters through a comprehensive analysis of reflectance in the angular domain. We investigated sensing parameters such as RI sensitivity, the reflectance minimum, and the figure of merit (FOM) as functions of the thickness of the silver layer, along with the refractive index of a coupling prism or the wavelength of a light source. To accomplish this, we developed an algorithm to analyze the optical response of the SPR structure, which was employed in the Kretschmann configuration for sensing aqueous solutions of NaCl. The algorithm was utilized to assess the sensing properties of the structure and to visualize the effect of certain varied parameters on the sensor’s performance, and surface plots were used to provide a better understanding of the sensor’s behavior.

To demonstrate the advantages of the use of 2D material, two types of structures were considered. As a reference, a simple structure (bare silver without any overlayer) was analyzed. Subsequently, a modified structure with the addition of 2D material, namely two monolayers of BP, was studied when surface plasmon polaritons (SPPs) propagated along two principal directions—zigzag (ZZ) and armchair (AC) directions. This aspect has often been omitted from research [23,25,28,35]. First, the reference structure was investigated at a wavelength of 632.8 nm as a function of the thickness of the silver layer together with the RI of a coupling prism, giving a silver layer thickness of 50 nm and a fused silica coupling prism. The achieved RI sensitivity ranged from 193.9 to 251.5 degrees per RI unit (deg/RIU), with the highest FOM reaching 52.3 RIU^−1^. Next, the modified structure with the addition of two monolayers of BP was optimized as a function of the thickness of the silver layer and the source wavelength. The achieved RI sensitivity for BP was in the range of 269.1–351.2 deg/RIU at the optimized wavelength of 628 nm, with the highest FOM reaching 44.7 RIU^−1^ for the ZZ direction.

The innovative approach of adjusting the source wavelength using a 1DPhC combined with an LED and the results of the optimization of the proposed SPR structure indicate its potential for the development of more sensitive and efficient employing a simple plasmonic structure.

## 2. Structure Characterization

**Figure 1:**
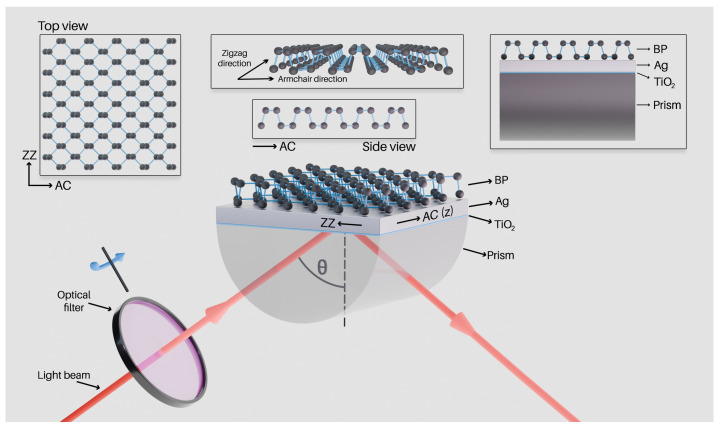
A schematic drawing of the SPR structure with a BP overlayer paired with an optical filter (the AC and ZZ directions of the BP are also outlined). The z-axis is parallel with the AC direction. The titanium dioxide adhesion layer is shown in teal, and the silver plasmonic layer is shown in gray. The incident optical wave is *p*-polarized, and the visualization is related to the sensor’s response for the ZZ direction.

This particular section covers all the dispersion formulas that were used to simulate the optical response of the proposed structure. The structure under study consists of a glass substrate, an adhesion layer of titanium dioxide, a silver layer, and a 2D material—black phosphorus. The structure is presented schematically in Figure 1.

### 2.1. Silver

The silver film supports the propagation of surface plasmon polariton (SPP) in the visible spectral range, and has advantages such as low losses and no interstate transitions [2]. The dispersion of the silver can be described by the complex dielectric function given by the Drude-Lorentz model [46]:

(1) $$\varepsilon_{\mathrm{Ag}}\left(\lambda \right)=\varepsilon_{\infty }-\frac{1}{\lambda_{p}^{2}\left(1/\lambda^{2}+i/\gamma_{p}\lambda \right)}-\sum_{j=1}^{n}\frac{A_{j}}{\lambda_{j}^{2}\left(1/\lambda^{2}-1/\lambda_{j}^{2}\right)+i\lambda_{j}^{2}/\gamma_{j}\lambda },$$

with the parameters listed in [46].

### 2.2. Fused Silica, Titanium Dioxide

Both the prism and substrate are supposed to be made of fused silica, and titanium dioxide comprises the adhesion layer. The refractive index of all the media as a function of wavelength can be described by the Sellmeier formula [47]:

(2) $$n^{2}\left(\lambda \right)=C+\sum_{i=1}^{n}\frac{A_{i}\lambda^{2}}{\lambda^{2}-B_{i}},$$

where λ is the wavelength in μm and the values of the Sellmeier coefficients for fused silica at room temperature are [47] *C* = 1; $A_{1}$ = 0.6961663; $A_{2}$ = 0.4079426; $A_{3}$ = 0.8974794; $B_{1}$ = 0.0684043 μm^2^; $B_{2}$ = 0.1162414 μm^2^; $B_{3}$ = 9.896161 μm^2^. Similarly, for the titanium dioxide, the values of the Sellmeier coefficients at room temperature are *C* = 2.7655; $A_{1}$ = 2.2; and $B_{1}$ = 0.26524 μm^2^.

### 2.3. Black Phosphorus

As shown in Figure 1, BP is an anisotropic material with two principal directions—the AC and ZZ directions [37,48,49,50,51,52]. Its optical properties can be deduced from rigorous analysis using a permittivity tensor [53,54], and the real and imaginary parts of its refractive indices along the AC and ZZ directions can be determined using the Cauchy absorption model [51,52]:

(3) $$n\left(\lambda \right)=A+B\frac{10^{4}}{\lambda^{2}}+C\frac{10^{9}}{\lambda^{4}},$$

(4) $$\kappa \left(\lambda \right)=D\times 10^{-5}+E\frac{10^{4}}{\lambda^{2}}+F\frac{10^{9}}{\lambda^{4}},$$

where λ is the wavelength in nm, and the Cauchy coefficients for the ZZ direction, which are taken from [52], are A=3.57; B=6.79 nm^2^; C=39.99 nm^4^; D=3206; E=−0.521 nm^2^; and F=10.26 nm^4^. For the AC direction, the coefficients are A=3.48; B=6.76 nm^2^; C=35.48 nm^4^; D=32780; E=−10.98 nm^2^; and F=50.71 nm^4^.

## 3. Methods

Achieving the optimal performance of an SPR sensor for specific analyte sensing requires consideration of a wide range of parameters. For this purpose, an algorithm was developed to analyze the optical response of a simple SPR structure, where the primary objective was to keep certain parameters fixed while varying others to observe the impact of the change on the sensor’s performance. This method required a thorough analysis of reflectance responses, examining thousands of resonance dips in the angular domain to reveal the dependence of sensing properties on varied parameters. In this study, the RI sensitivity $S_{n}$, the reflectance minimum $R_{p,\min}$, and the FOM were investigated as a function of the thickness of the silver layer $t_{\mathrm{Ag}}$ together with either the refractive index of a coupling prism, $n_{p}$, or the wavelength, λ, of a light source. The RI sensitivity can be defined as

(5) $$S_{n}=\frac{\delta \theta_{r}}{\delta n},$$

where $\delta \theta_{r}$ is the change in the resonant angle, which corresponds to the shift in the position of the dip related to the change in the refractive index of the analyte, δn.

In addition to the RI sensitivity, it is essential to also consider the FOM. The depth of the dip *D*, which is associated with the minimum reflectance, is a crucial factor in determining the FOM. Therefore, it is necessary to take this factor into account. The expanded definition of the FOM is the following [55]:

(6) $$\mathrm{FOM}=D\frac{S_{n}}{\mathrm{FWHM}},$$

where the FWHM is a full-width half maximum of the resonance dip.

**Figure 2:**
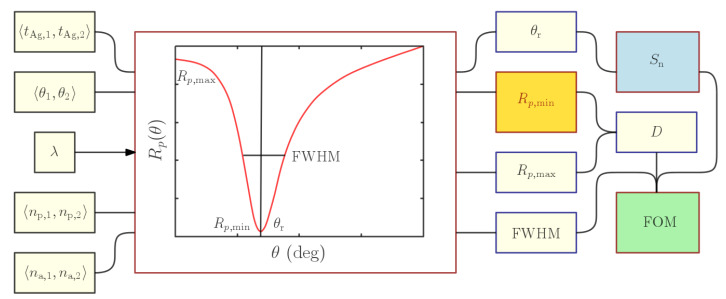
Schematic drawing of the algorithm.

The layout of the algorithm used to analyze the sensing properties of the structure in the angular domain is depicted in Figure 2. It required a set of input parameters, such as the thickness of the silver layer, the refractive index of a coupling prism, the refractive index of the analyte, and the source wavelength. As mentioned previously, the main goal was to keep certain parameters constant while altering others to observe their impact on the sensing parameters of the structure. This was accomplished by analyzing the reflectance responses, where various output parameters were gathered, such as the resonance angle $\theta_{r}$, reflectance minimum $R_{p,\min}$, reflectance maximum $R_{p,\max}$, and FWHM for each resonance dip.

The optical response of the SPR structure was modeled using the transfer matrix method [56]. The algorithm aimed to extract the dip parameters calculated for specific input parameters. The algorithm consisted of several steps, each executed by a specific MATLAB script. Initially, the reflectance responses for a range of angles of incidence $<\theta_{1},\theta_{2}>$, were calculated for all refractive indices of the analyte within a given interval, $<n_{a,1},n_{a,2}>$, for the single silver layer thickness $t_{\mathrm{Ag}}$ and the refractive index of the coupling prism, $n_{p}$. The calculated reflectance responses for a specific wavelength λ, were then examined individually to obtain the value and position of $R_{p,\min}$ in the $n_{p}-t_{\mathrm{Ag}}$ plane. Therefore, the values of other output parameters such as $\theta_{r}$, $R_{p,\max}$ or the FWHM were obtained similarly and were stored in a data sheet. This sheet was simultaneously accessed by follow-up scripts that, based on the output parameters, determined the RI sensitivity and FOM. The mentioned process was then repeated for all combinations of silver layer thicknesses in the interval $<t_{\mathrm{Ag},1},t_{\mathrm{Ag},2}>$ and the refractive indices of the coupling prism $<n_{p,1},n_{p,2}>$. To gain insight into the sensor’s behavior, surface plots could be used to analyze the data sets and optimize the sensor’s performance.

## 4. Results and Discussion

The output parameters could be utilized to assess the sensing properties of the structure. As a result, it became possible to visualize the effect of certain varied parameters on the sensor’s performance, leading to a quick understanding of the sensor’s behavior. To conduct this theoretical analysis, the scripts, written in MATLAB 2020a software, were run on the following PC configuration: AMD RYZEN 7 2700, 16 GB (RAM), and MSI GeForce GTX 1660 Ti X 6G (GPU).

### 4.1. Prism Selection

Optimization had to be performed for specific applications of the sensor. In this case, aqueous solutions of NaCl with varying mass concentrations of NaCl in water ranging from 0%wt to 10%wt with a step of 2%wt were considered analytes. The corresponding analyte refractive indices at a wavelength of 589.3 nm were 1.3330, 1.3360, 1.3391, 1.3421, 1.3451, and 1.3482. Initially, the analysis was focused on the selection of a coupling prism, and the RI sensitivity $S_{n}$, reflectance minimum $R_{p,\min}$, and FOM as a function of the silver layer thickness together with the refractive index of the coupling prism at a wavelength of 632.8 nm are shown in Supplementary Figure S1a–f.

The considered interval of the refractive index of the coupling prism ranged from 1.45 to 1.75 while simultaneously the silver layer thickness was varied in the range from 40 to 60 nm. The RI of the analyte ranged from 1.3330 to 1.3482, and the surface plots shown in Supplementary Figure S1a–f are depicted for the lowest and the highest part of the considered RI interval.

It is evident from Supplementary Figure S1a,b that the ideal coupling prism in terms of RI sensitivity had to have a lower refractive index than that of standardly used BK7 or SF10 glass. Moreover, as shown in Supplementary Figure S1c,d, the most pronounced resonance dips could be achieved for the silver layer thickness ranging from 45 nm to 50 nm. In addition, the results shown in Supplementary Figure S1e,f provide the information about the highest achievable FOM. The structure with the silver layer thickness of 50 nm was chosen together with fused silica as the ideal coupling prism.

It is important to note that in this multicriteria optimization, it was not possible to design a structure that would have the highest RI sensitivity and achieve the highest FOM at the same time, as clearly seen from the surface plots presented in Supplementary Figure S1a,b,e,f. Additionally, the optimal parameters depended on the analyte RI behavior. The larger the difference between the lowest and highest values of the RI of the sensed medium, the greater the difference in the optimal parameters of the structure.

**Figure 3:**
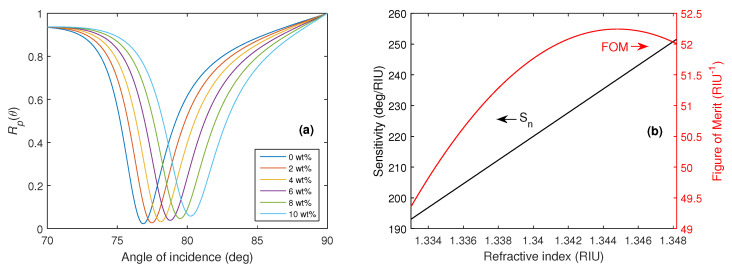
The theoretical angular reflectance $R_{p}\left(\theta \right)$ when aqueous solutions of 0–10 wt% NaCl in water were considered analytes (**a**) and the corresponding RI sensitivity $S_{n}$, together with FOM (**b**).

To demonstrate the sensing properties of the structure for the optimized silver layer thickness $t_{\mathrm{Ag}}=50$ nm and fused silica as the coupling prism at a wavelength of 632.8 nm, the reflectance $R_{p}\left(\theta \right)$ as a function of the angle of incidence θ together with the corresponding RI sensitivity $S_{n}$ and FOM are shown in Figure 3a,b respectively.

**Figure 4:**
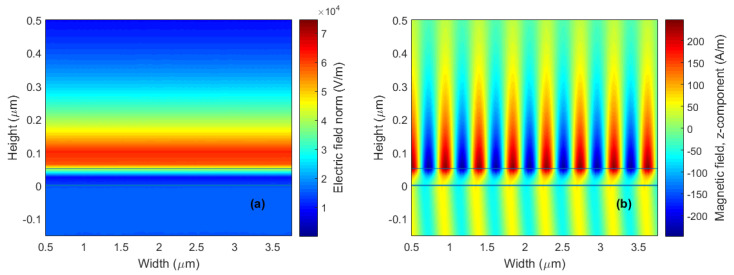
The electric field magnitude (**a**) and the tangential component of the magnetic field (**b**) in the structure at a wavelength of 632.8 nm when distilled water was considered an analyte for the angle of incidence $\theta =76.85^{\circ }$.

The achieved RI sensitivity $S_{n}$ for the reference structure varies in a range of 193.9–251.5 deg/RIU and the highest FOM attains a value of 52.3 RIU^−1^. A more detailed description of the SPR effect at the analyte-metal interface can be provided by depicting the electric and magnetic field distributions, which are shown in Figure 4a,b at the resonance angle $\theta_{r}=76.85^{\circ }$ when distilled water is considered as an analyte.

### 4.2. Black Phosphorus

Amongst the 2D materials that led to the enhancement of the sensing properties of the SPR structure, BP stood out as the most effective. In parallel with the previously demonstrated approach, we started with different numbers of BP monolayers, and the auxiliary analysis of the BP monolayer count showed that the best approach was using two monolayers of BP. Further increase in the BP monolayer count for the considered analyte RI range would have negatively affected the sensor response, thus worsening its performance. The resonance dips became shallower and wider and shifted less with the analyte RI change, as further explained below in the supplementary surface plots. Due to BP being an in-plane anisotropic material, the sensor’s response was studied considering SPPs propagating in the different principal AC and ZZ directions as illustrated in Figure 1. Therefore, the analysis could be further expanded by the addition of two monolayers of BP to the SPR structure with a monolayer thickness of $t_{\mathrm{bp}}=0.53$ nm. For computation purposes, two sheets of BP were represented by a single layer of anisotropic material with thickness $t_{\mathrm{bp}}=1.2$ nm [57].

Regarding the source wavelength λ, it was possible to utilize a highly accessible LED source along with a 1DPhC with a defect layer [45] as a tunable optical filter [58], allowing the use of a variable-wavelength LED-based source with a narrow linewidth. The employed crystal consisted of alternating layers of titanium dioxide (TiO_2_) and silicon dioxide (SiO_2_) with a defect layer of SiO_2_ with a greater thickness, which served as an optical cavity that supported defect mode excitation. The defect mode resonance could be tuned by the adjustment of the angle of incidence of light on the 1DPhC. Therefore, by tilting the crystal as shown in Figure 1, the wavelength of the source could be easily adjusted.

This innovative approach can lead to a significant increase in the RI sensitivity $S_{n}$ and an improvement in the reflectance minimum $R_{p,\min}$ and FOM. These parameters as a function of the silver layer thickness $t_{\mathrm{Ag}}$ together with the source wavelength λ, are shown in Supplementary Figure S2a–f for the AC direction and in Supplementary Figure S3a–f for the ZZ direction.

Furthermore, for the AC direction, it is evident in Supplementary Figure S2a,b that the highest RI sensitivity, indicated by the deep red color, shifts from the lower-right corner to the central part of the $\lambda -t_{\mathrm{Ag}}$ plane. Adding another BP monolayer would further shift the sensitivity towards the upper left corner, justifying the use of two monolayers of BP as the best option for sensing the chosen analyte. Additionally, the same behavior can be seen in Supplementary Figure S2c,d, where it is evident that the dips would become more shallow, thus resulting in a lower FOM, as depicted in Supplementary Figure S2e,f. Similar behavior for the above mentioned characteristics can also be observed for the ZZ direction in Supplementary Figure S3a–f.

**Figure 5:**
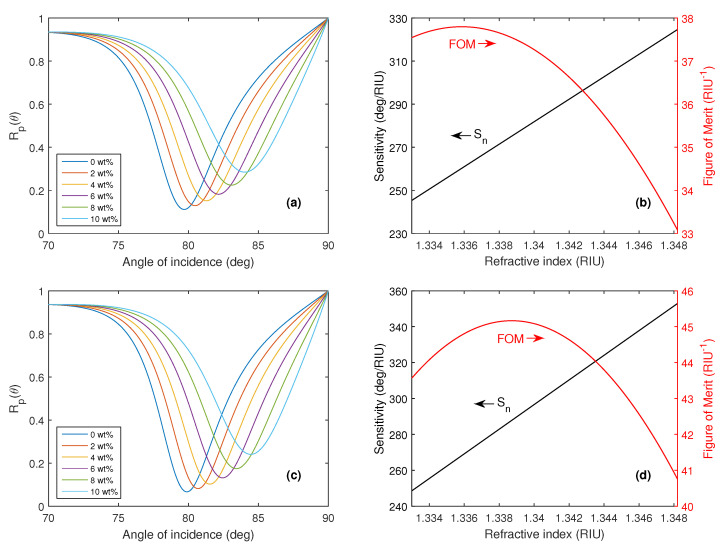
The theoretical angular reflectance $R_{p}\left(\theta \right)$ when aqueous solutions of 0–10 wt% NaCl in water were considered analytes and the corresponding RI sensitivity together with FOM for the structure with two monolayers of BP in the AC direction (**a**,**b**) and ZZ direction (**c**,**d**).

To demonstrate the sensing capabilities of the SPR structure with two monolayers of BP for both the AC and ZZ directions, an optimized silver layer thickness of 50 nm and a wavelength of 633 nm were chosen, and the reflectance $R_{p}\left(\theta \right)$ as a function of the angle of incidence θ, together with the corresponding RI sensitivity $S_{n}$ and FOM are shown in Figure 5a,c and Figure 5b,d, respectively. The achieved RI sensitivity $S_{n}$, for the structures with two monolayers of BP in the AC direction and ZZ direction varied in the range of 245.2–324.5 deg/RIU and 248.4–352.7 deg/RIU respectively, and the highest FOM values were 37.8 RIU^−1^ and 45.1 RIU^−1^, respectively. The expansion of the SPR structure using two monolayers of BP led to a significant increase in RI sensitivity, with a slight decrease in the FOM.

**Figure 6:**
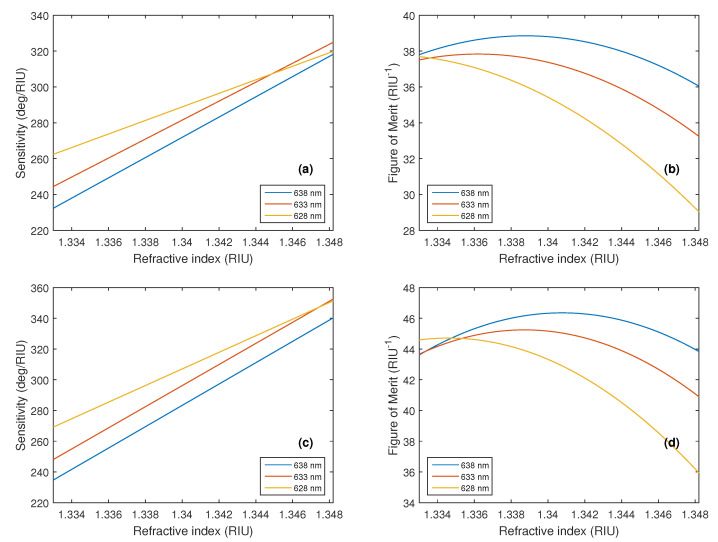
A comparison of the theoretical RI sensitivity $S_{n}$ and figure of merit as a function of the refractive index *n* of the analyte for different source wavelengths for the structure with two monolayers of BP in the AC direction (**a**,**b**) and ZZ direction (**c**,**d**).

A comparison of the RI sensitivity $S_{n}$ and FOM for different source wavelengths for the structure with two monolayers of BP is shown in Figure 6a,c and Figure 6b,d respectively. The enhanced sensitivity with the silver layer thickness of 50 nm and the optimized wavelength of 628 nm varied in the range of 269.1–351.2 deg/RIU, with the highest FOM value being 44.7 RIU^−1^ for the ZZ direction.

**Figure 7:**
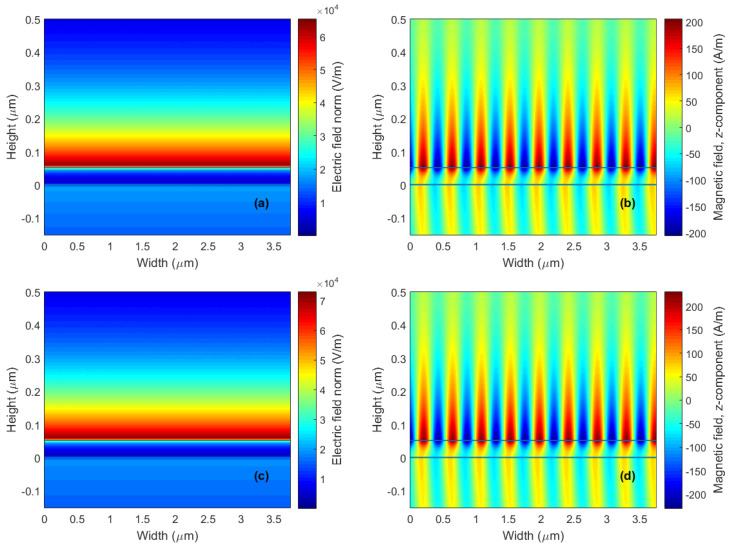
The electric field magnitude and the tangential component of the magnetic field in the structure with two monolayers of BP in the AC direction ($\theta =79.31^{\circ }$; (**a**,**b**)) and the ZZ direction ($\theta =79.47^{\circ }$; (**c**,**d**)) at a wavelength of 632.8 nm when distilled water was considered an analyte.

Furthermore, the electric and magnetic field distributions in the structure with two monolayers of BP for both directions at a wavelength of 632.8 nm are shown in Figure 7a–d when distilled water was considered an analyte. The RI sensitivity increase for different sensing structures (from a bare silver to BP in AC or ZZ directions) is demonstrated by the enhancement in the magnitude of electric field intensity. Analysis of the enhancement of electric field intensity and thus sensitivity increase could be extended to sensors employing monolayers of other 2D materials such as graphene, MoS_2_, etc. It was revealed that the BP-based sensors were characterized by the greatest field enhancement, which demonstrated their superiority over available 2D materials. In addition, the large specific area of BP extends its potential in providing a highly sensitive, fast and label-free detection method for several biomolecules such as disease biomarkers.

A comparison with other 2D material-based SPR sensor structures utilizing a silver plasmonic layer is presented in Table 1. It is clear that the simple structure exhibited a significant increase in RI sensitivity due to the incorporation of 2D material, optimized parameters, and source wavelength adjustment, which was demonstrated using an innovative approach. While some structures may demonstrate a higher sensitivity or FOM compared to the proposed design, their complexity often makes them more challenging to manufacture, such as in matching the lattice parameters of 2D materials. In contrast, combining structural simplicity with parameter optimization can yield comparable results. In the 2D material-based SPR sensors listed in Table 1, the nearest to our design is a structure employing the 2D materials of BP and WS_2_ with the use of a coupling prism of CaF_2_ [59]. In this case, both high RI sensitivity and FOM are achieved simultaneously. Additionally, adjusting the source wavelength can further enhance the sensor’s performance. This wavelength tunability allows for increased sensitivity across the entire range of the analyte RI and is a significant advantage since it does not require any modifications to the sensing structure.

## 5. Conclusions

In this paper, a comprehensive analysis of reflectance responses in the angular domain was presented for a simple SPR structure including a silver layer. The silver layer thickness, together with the RI of a coupling prism and the source wavelength, was varied to obtain insights into the behavior of the sensor and enable the optimization of its performance. This approach was taken for aqueous solutions of NaCl based on RI sensitivity, the reflectance minimum, and the FOM. Additionally, the analysis was extended to the SPR structure’s modification with 2D material, two monolayers of BP, for which SPP propagation along ZZ and AC directions was considered.

For the selected structure, the optimization gave a wavelength of 632.8 nm, a thickness of 50 nm for the silver layer, and a fused silica coupling prism. The achieved RI sensitivity varied in a range of 193.9–251.5 deg/RIU, with the highest FOM value being 52.3 RIU^−1^. The optimization was extended to a similar structure with the addition of two monolayers of BP in both the AC and ZZ directions. The optimization results included a thickness of 50 nm for the silver layer and the achieved RI sensitivity, which varied in the range of 269.1–351.2 deg/RIU at the optimized wavelength of 628 nm, with the highest FOM reaching 44.7 RIU^−1^ for the ZZ direction.

The simple plasmonic structure design is advantageous from a manufacturing standpoint. More complex structures with an increasing number of degrees of freedom may be affected by manufacturing tolerances. The results of the optimization indicate that the innovative approach of adjusting the wavelength of a source using a 1DPhC including a defect layer combined with an LED has potential in the development of efficient and sensitive sensors employing a simple plasmonic structure.

## Figures and Tables

**Table 1:** Angular interrogation-based sensor performance comparison.

Structure	Wavelength (nm)	Sensitivity (deg/RIU)	FOM (RIU^−1^)	Reference
BK7/Ag/BP/Graphene	633	217	–	[35]
BK7/Ag/BP/WSe_2_	633	279	–	[35]
BAK1/Ag/WS_2_/FASnI_3_/BP	633	402	48.2	[60]
BK7/ZnO/Ag/BaTiO_3_/WS_2_	633	235	63.51	[61]
BK7/ZnO/Ag/BaTiO_3_/MoS_2_	633	195	20.67	[61]
BK7/ZnO/Ag/BaTiO_3_/Graphene	633	168	58.33	[61]
BK7/Ag/Graphene	457.5	300.26	33.25	[62]
CaF_2_/TiO_2_/Ag/PtSe_2_/WS_2_	632.8	240.1	78.46	[63]
BK7/Ag/TiO_2_/Graphene	480	302.26	33.76	[64]
CaF_2_/Ag/BP/WS_2_	633	375	65.78	[59]
BK7/Ag/Au/BaTiO_3_/Graphene	633	294	42.13	[65]
BK7/Ag/WS_2_/BaTiO_3_/BP	633	370	60	[66]
BK7/Ag/12 layers BP (AC)	633	211.1	–	[67]
BK7/Ag/12 layers BP (ZZ)	633	287.9	–	[67]
FS/TiO_2_/Ag	632.8	251.5	52.3	This work
FS/TiO_2_/Ag/BP (AC)	633	324.5	37.8	This work
FS/TiO_2_/Ag/BP (ZZ)	628	351.2	44.7	This work

## Data Availability

The data presented in this study are available upon reasonable request from the corresponding authors.

## Supplementary Materials

The following supporting information can be downloaded at: https://www.mdpi.com/article/10.3390/nano15110790/s1, Supplementary Figure S1: Theoretical RI sensitivity, reflectance minimum and figure of merit for distilled water (a,c,e), 10 wt% of NaCl in water (b,d,f) for the reference structure; Supplementary Figure S2: Theoretical RI sensitivity, reflectance minimum and figure of merit for distilled water (a,c,e), 10 wt% of NaCl in water (b,d,f) for the structure with two monolayers of BP (plane of incidence coincides with the AC direction); Supplementary Figure S3: Theoretical RI sensitivity, reflectance minimum and figure of merit for distilled water (a,c,e), 10 wt% of NaCl in water (b,d,f) for the structure with two monolayers of BP (plane of incidence coincides with the ZZ direction).
