# Supplementary material for: Thickness and Wavelength Optimizations of a High-Performance SPR Sensor Employing a Silver Layer and Black Phosphorus in Principal Directions
Source: Nanomaterials (Basel). 2025 May 24;15(11):790. doi: 10.3390/nano15110790 (PMC12157747; doi:10.3390/nano15110790)
Supplement: Supplementary file 1 [file nanomaterials-15-00790-s001.zip › nanomaterials-3637225-supplementary.pdf]

Thickness and wavelength optimizations of a high-performance SPR sensor employing a silver layer and black phosphorus in principal directions

Jakub Chylek , Dalibor Ciprian , and Petr Hlubina*

Department of Physics, Technical University Ostrava, 17. listopadu 2172/15, 708 00 Ostrava-Poruba, Czech Republic
*Correspondence: petr.hlubina@vsb.cz (P.H.)

Supplementary Materials

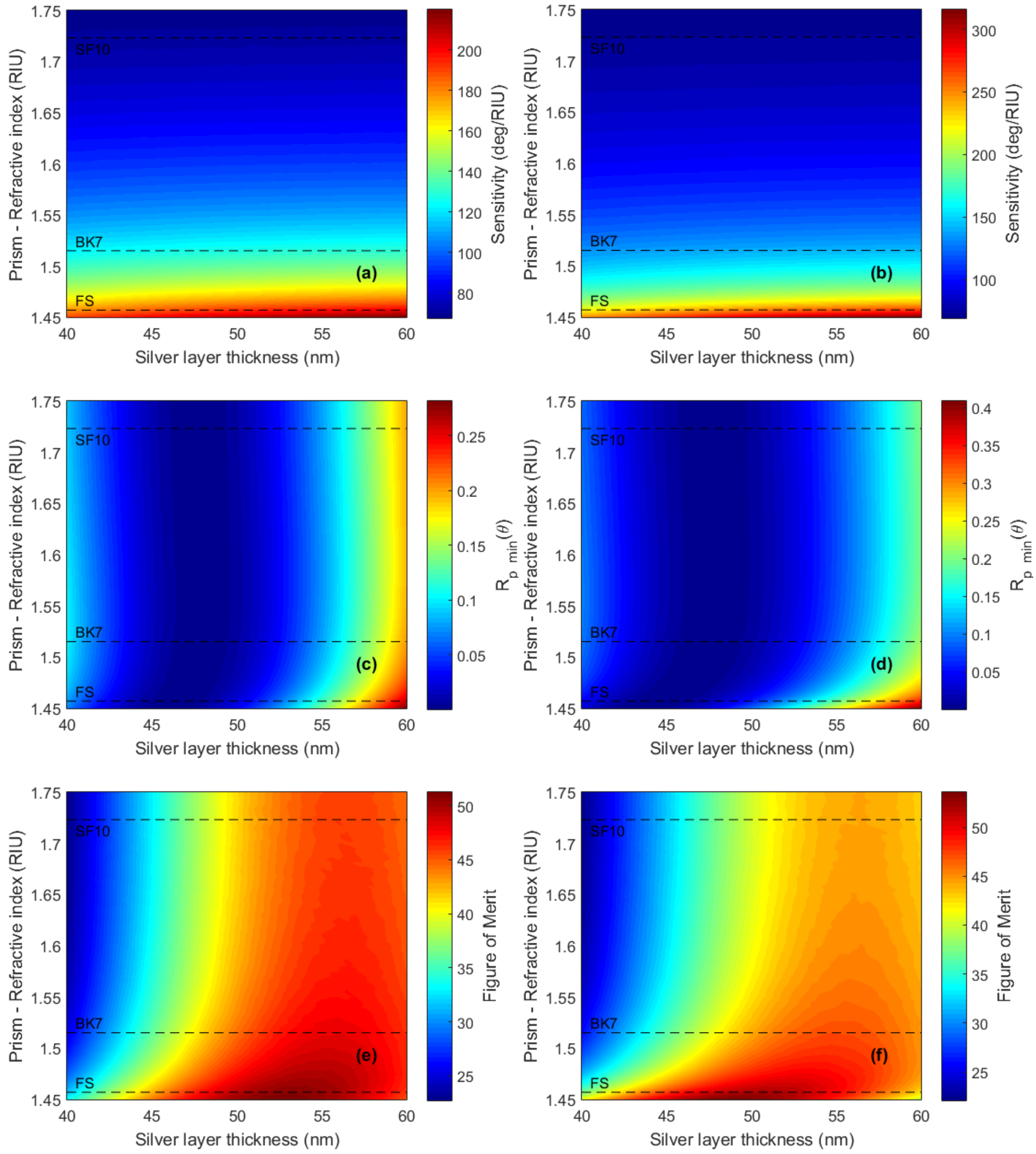

Supplementary Figure S1. Theoretical RI sensitivity, reflectance minimum and figure of merit for distilled water (a), (c) and (e), 10 wt% of NaCl in water (b), (d) and (f) for the reference structure.

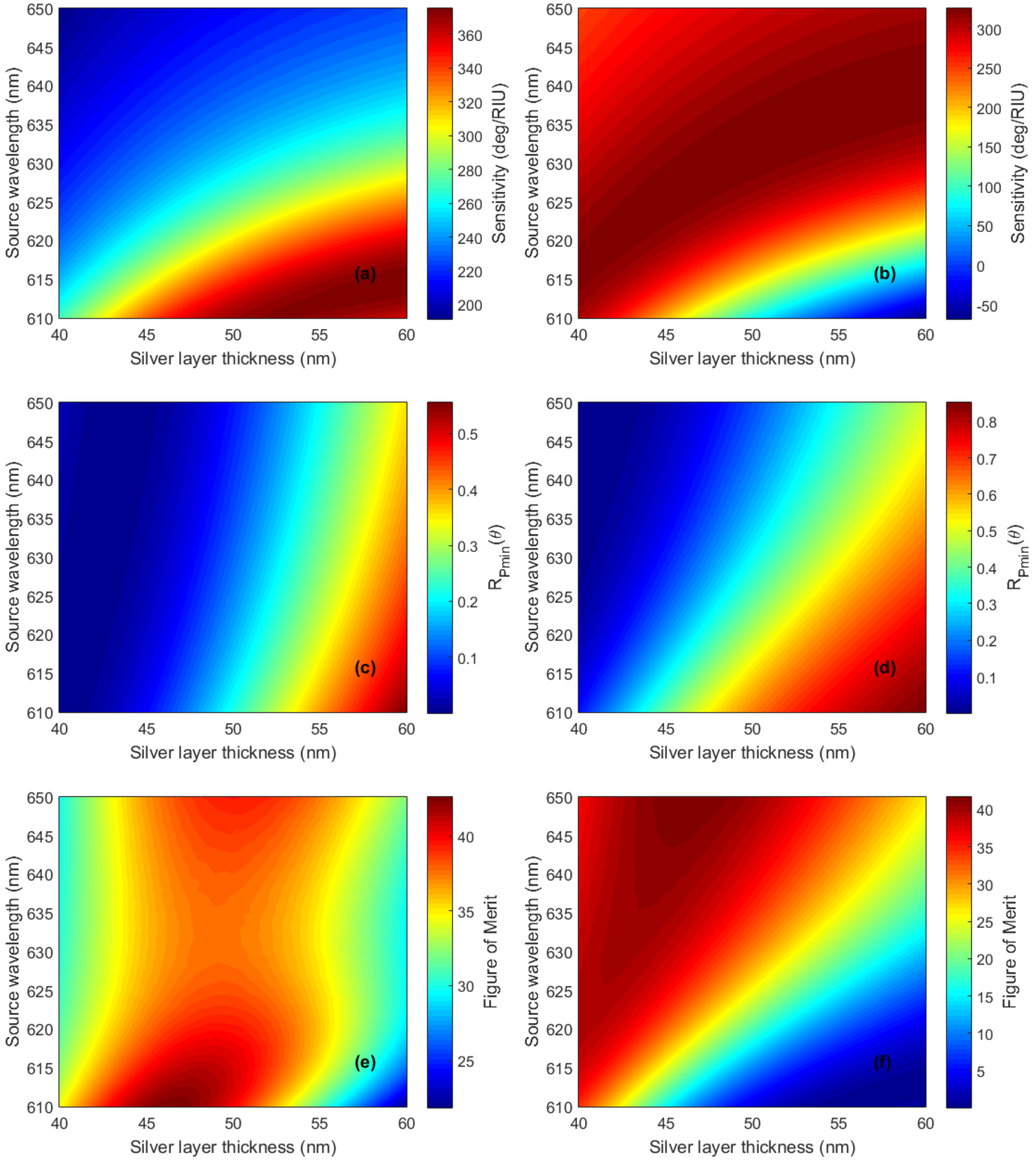

Supplementary Figure S2. Theoretical RI sensitivity, reflectance minimum and figure of merit for distilled water (a), (c) and (e), 10 wt% of NaCl in water (b), (d) and (f) for the structure with two monolayers of BP (plane of incidence coincides with the AC direction).

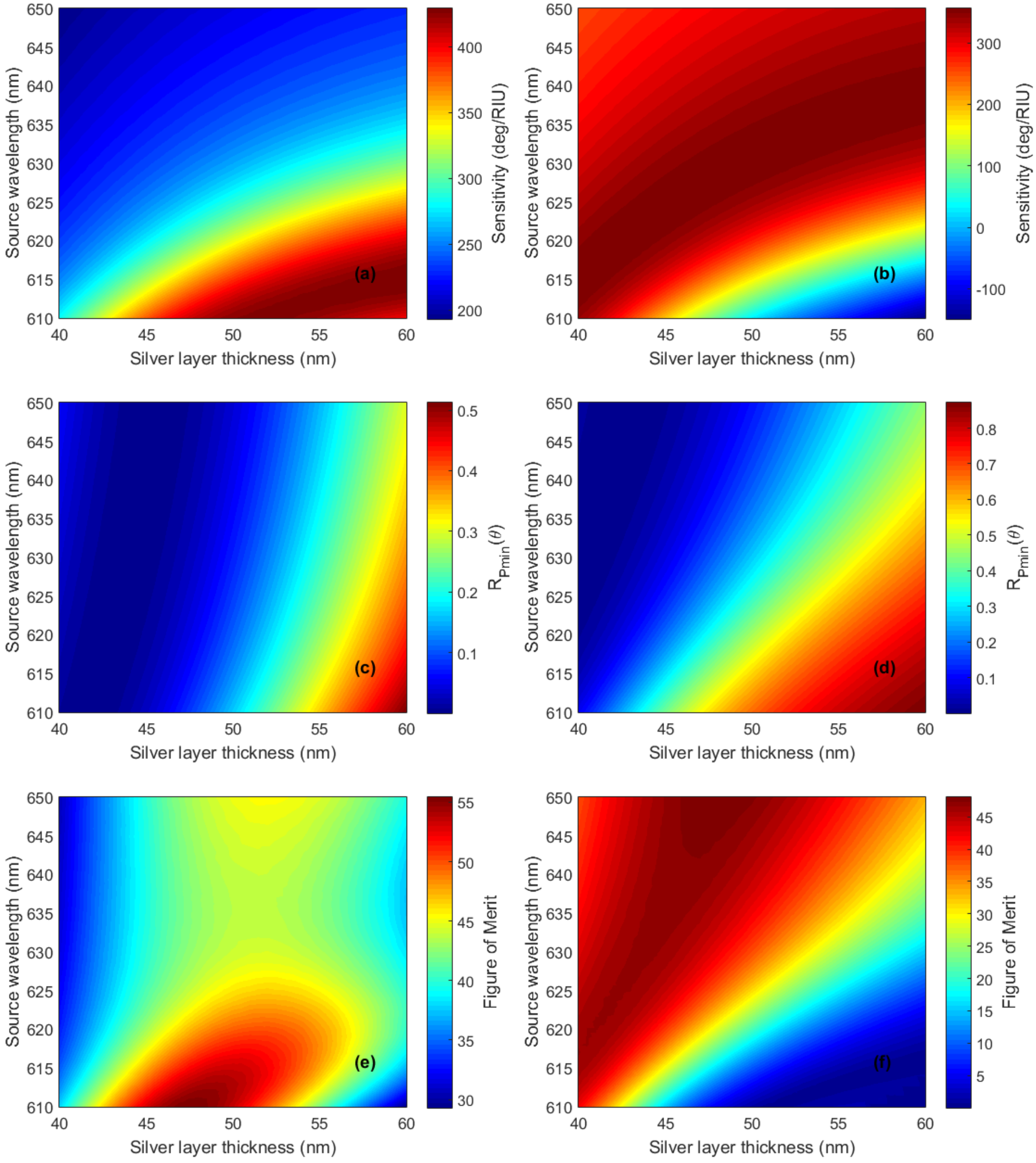

Supplementary Figure S3. Theoretical RI sensitivity, reflectance minimum and figure of merit for distilled water (a), (c) and (e), 10 wt% of NaCl in water (b), (d) and (f) for the structure with two monolayers of BP (plane of incidence coincides with the ZZ direction).
